# 3D Road Lane Classification with Improved Texture Patterns and Optimized Deep Classifier

**DOI:** 10.3390/s23115358

**Published:** 2023-06-05

**Authors:** Bhavithra Janakiraman, Sathiyapriya Shanmugam, Rocío Pérez de Prado, Marcin Wozniak

**Affiliations:** 1 Department of Computer Science and Engineering, Dr. Mahalingam College of Engineering and Technology, Pollachi 642003, India; 2 Department of Electronics and Communication Engineering, Panimalar Engineering College, Chennai 600123, India; 3 Telecommunication Engineering Department, University of Jaén, 23700 Linares, Spain; 4 Faculty of Applied Mathematics, Silesian University of Technology, 44-100 Gliwice, Poland

**Keywords:** bidirectional gated recurrent unit, local Gabor binary pattern histogram sequence, local texton XOR pattern, median ternary pattern, road lane classification, self-improved honey badger optimization

## Abstract

The understanding of roads and lanes incorporates identifying the level of the road, the position and count of lanes, and ending, splitting, and merging roads and lanes in highway, rural, and urban scenarios. Even though a large amount of progress has been made recently, this kind of understanding is ahead of the accomplishments of the present perceptual methods. Nowadays, 3D lane detection has become the trending research in autonomous vehicles, which shows an exact estimation of the 3D position of the drivable lanes. This work mainly aims at proposing a new technique with Phase I (road or non-road classification) and Phase II (lane or non-lane classification) with 3D images. Phase I: Initially, the features, such as the proposed local texton XOR pattern (LTXOR), local Gabor binary pattern histogram sequence (LGBPHS), and median ternary pattern (MTP), are derived. These features are subjected to the bidirectional gated recurrent unit (BI-GRU) that detects whether the object is road or non-road. Phase II: Similar features in Phase I are further classified using the optimized BI-GRU, where the weights are chosen optimally via self-improved honey badger optimization (SI-HBO). As a result, the system can be identified, and whether it is lane-related or not. Particularly, the proposed BI-GRU + SI-HBO obtained a higher precision of 0.946 for db 1. Furthermore, the best-case accuracy for the BI-GRU + SI-HBO was 0.928, which was better compared with honey badger optimization. Finally, the development of SI-HBO was proven to be better than the others.

## 1. Introduction

Transportation is becoming an essential part of contemporary society’s everyday routine. As the number of vehicles has rapidly increased in recent decades, the frequency of traffic accidents and the provocation of traffic jamming have become increasingly noticeable [1,2,3]. The entire country suffers tremendous economic losses as a result of road traffic accidents, which pose a major threat to people’s safety. In this situation, more and more drivers are opting for the lane maintenance system [4,5,6]. In a lane-keeping system, lane line recognition and lane offset estimation are strongly tied to the implementation of the lane-keeping function, which has a direct impact on the system’s resilience and real-time performance [6,7].

Road lane recognition using image processing and machine learning approaches has been a popular topic of study in both the advanced and developing worlds [8,9,10]. Since the number of vehicles has increased, several clever technologies have been developed to assist drivers in driving safely. Lane recognition is a significant feature of any driver assistance system. Scientists working on lane detection are now confronting numerous important obstacles, including achieving dependability in the face of sunlight and backdrop clutter [11,12]. The advancement of image processing methods and the development of cheap visual sensor devices have opened the way for a variety of autonomous road lane recognition approaches in recent years. The actuality that the textures are alike makes automated road-lane-detecting techniques feasible. The fact that the textures of lanes are distinguishable from the background of the concrete surface makes automatic road lane recognition techniques feasible [13,14,15]. 

Due to the following scenarios, lane detection becomes difficult: With less understanding of the road geometry, lane-marking alternatives develop the presence of surrounding impediments, which obscure lane markings or are misinterpreted as lane markings. On-road signage or writing, as well as shadow effects, are frequently recommended as lane feature locations. The variation in illumination affects color and intensity. Several specialists have approved lane detection algorithms in the previous two decades. Feature-based or model-based approaches are used in traditional lane-detecting systems [16,17]. To extract lane line information, the feature-oriented method primarily employs the color and gradient variation in lane lines. The detection quality of the lane line identification schemes that depend on conventional techniques is easily influenced by climate changes owing to the limitations of the feature extraction technique [18]. When the assumption of flat ground is violated, conventional algorithms become inaccurate while recognizing lanes in the image domain and projecting them into the 3D environment in both elevation and lane curvature. Based on the recent success of CNNs in detecting lanes in 3D with the monocular depth estimation, more models are aiming to produce a series of 3D curves in camera coordinates from a single front-facing camera image, with each curve expressing either a lane delimiter or a lane center line. Moreover, the aforementioned approaches need lane line detection before calculating the lane offset for cars, the end-to-end lane offset estimation, which is not conceivable in the existing approaches. In order to overcome the above-stated limitations, the contributions are defined as follows:

1. The primary goal of this work is to propose a 3D road lane classification with improved texture patterns and an optimized deep classifier that includes Phases I (road or non-road classification) and II (lane or non-lane classification);
2. Initially, features such as the local texton XOR pattern (LTXOR), local Gabor binary pattern histogram sequence (LGBPHS), and median ternary pattern (MTP) are determined. These features are further classified using the bidirectional gated recurrent unit (BI-GRU), which determines whether there is a lane or not under various environmental conditions;
3. To improve its performance with the targets of lowering the complexity and minimizing the error, the weights in the BI-GRU are optimized by self-improved honey badger optimization (SI-HBO). Thus, the lane line problem in multi-lane scenes is efficiently recognized, and once the vehicles change lanes, the current lane scene is easily identified.

The structure of this study is specified as follows: Section 2 describes the existing research work. Section 3 explains the developed technique, and Section 4 describes the proposed features. Section 5 elaborates on the concept of two-phase classification. Section 6 presents the results and discussion and a comparative analysis. Finally, Section 7 explains the conclusion.

## 2. Literature Review 

In 2021, Satish et al. [19] proposed a novel strategy to alert the driver when the car crosses the road border lanes using machine learning methods to prevent road accidents and ensure safe driving. The dataset’s performance was measured by the production of experimental findings. The proposed technique outperformed the existing lane recognition algorithms regarding precision and accuracy. In 2021, Malik et al. [20] presented a CNN for lane offset estimation and lane line identification, which turned lane line detection difficulties into instance segmentation problems in a complicated road environment. The network builds its example for every line in response to changes in the lane process mechanism. The global scale perceptual optimization mechanism was intended to deal with the problem, particularly as the lane path length narrows as it approaches the vanishing point. In addition, an estimation network was employed to achieve multi-tasking processing and boost performance.

In 2020, Tiago et al. [21] presented a novel road representation as well as a workflow of processes for combining two concurrent DL schemes, based on two ENet model modifications. The findings revealed that the total solution copes with the many failures in every approach, resulting in a more reliable road detection result than each technique alone. In 2021, Ting et al. [22] used a two-level CNN to create an LCR model using visual technologies. To compare with the LCR approach, a new CNN based on the AlexNet was presented. For the two models, all samples were separated into a training dataset and testing dataset. Two machine networks were compared in terms of performance. With the LCR model, the average training accuracy was over 94.6 percent. The LCR model surpassed the AlexNet model, which averaged just a 73.97 percent accuracy.

In 2020, Jau et al. [23] built a DL-based embedded road-border-detecting system. A CAE with noise removal and reconstructing features was deployed to eliminate all items in the photos excluding lane markings to generate an image with clear lane markings. The lane line’s feature points were then retrieved, and a hyperbolic model was used to fit the lane line. Finally, for lane tracking, a particle filter was applied. According to the testing findings, the suggested lane-detecting method was 90.02 percent accurate for both structured and unstructured roadways. In 2019, Wang et al. [24] provided a straight-curve model-based curve identification technique, which has high application in most curved-road circumstances. By assessing the basic properties of the road images, the approach split the road image into the ROI and the road backdrop region. The ROI was further separated into two sections: straight and curved. Simultaneously, the mathematical formalism of a straight curve was constructed. Finally, the curve and straight detection and identification were accomplished, and the road lane line was recreated.

In 2021, Luo et al. [25] presented a unique and resilient multiple-lane recognition approach depending on road construction data that included five complementary specifications: length, parallel, distribution, pair, and uniform width. To choose lane candidates, all five limitations were merged into a cohesive framework based on HT. Furthermore, a dynamic programming technique to discover the most reasonable solutions from the remaining choices was provided. This technique may successfully cope with multi-lane detection’s combination of complexity and interferences. In 2022, Ye et al. [26] described a scheme to extract lane characteristics from MLS point clouds on curving highways. There were four phases in the suggested technique. A road edge recognition technology was used to discern road curbs and retrieve road surfaces following data pre-processing. Then, symbols, arrows, and phrases were discovered to alert drivers in critical situations. According to the research, the proposed approaches achieved greater accuracy and resilience than most current methods.

Table 1 reviews the work on road lane detection. Here, ref. [19] used a CNN that offered high accuracy and high precision. However, a cost analysis should be made. The CNN used in [20] undergoes less loss and reliable recall, but forward collision warning is not a concern. The ENet used in [21] achieves higher reliability and less scalability, but a special network is required for fusing purposes. The LCR used in [22] offers a high detection rate and high accuracy. However, it requires the use of more datasets. Moreover, ref. [24] used a particle filter, which provided less error with high accuracy. However, the collision-avoiding model needs to be involved. In [24], an improved Hough transform was used, in which a higher accuracy and minimal time utilization were established. However, a cost analysis should be made. The Hough transform used in [25] provided higher accuracy and fewer false alarms. However, it needs more consideration of the time usage. The Gaussian distribution used in [26] offered high precision and a high F1-score. However, the spiral curves of roads were not considered.

The aforementioned technique, however, does give a general concept as to how deep learning technology might be used to identify lane lines, but it is still unable to resolve end-to-end lane line detection in challenging environments. In spite of the challenging circumstances of lane line occlusion, high exposure, and unstructured roads, there are not many academics working on lane line identification at the moment. Additionally, the end-to-end lane offset estimation was not achieved in any of the aforementioned methods because they all used lane line detection before calculating the lane offset for cars. To overcome all these challenges, the proposed method can be used. 

## 3. Description of Proposed Technique 

This work developed Phase I (road or non-road classification) and Phase II (lane or non-lane classification) technology with 3D images. Initially, a 3D image is converted to a 2D image. Then, the detection process proceeds:

- At first, the proposed LTXOR-, LGBPHS-, and MTP-based features are extracted;
- Then, road or non-road is classified using the BI-GRU model;
- Further, these extracted features are provided for the optimal BI-GRU for lane or non-lane classification;
- For optimizing the weights in the BI-GRU, SI-HBO is deployed in this work.

**Figure 1:**
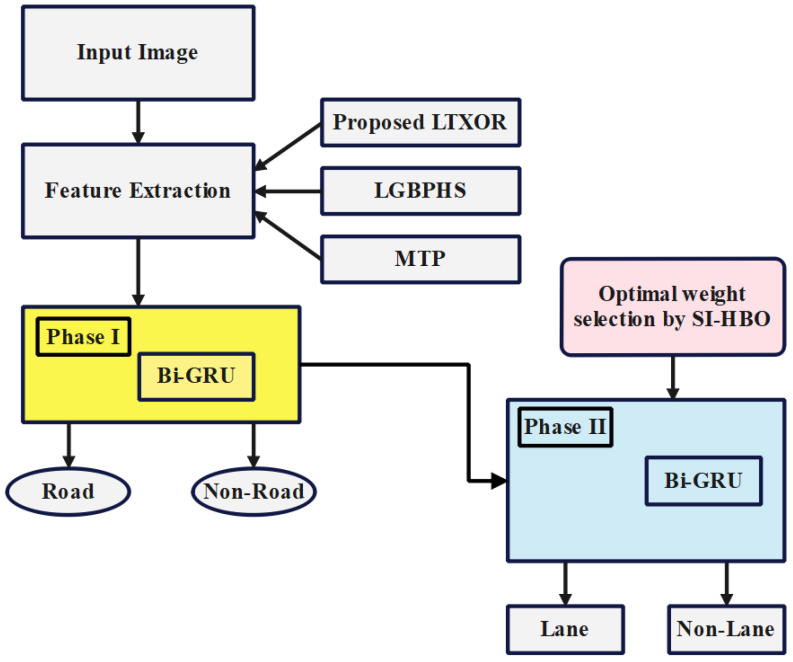
Overall architecture of the proposed model for road lane classification.

The overall architecture of the proposed model on road lane classification is shown in Figure 1.

## 4. Extraction of Proposed Features 

The proposed LTXOR-, LGBPHS-, MTP-based features are derived from the input image ($I_{im}$). 

### 4.1. Proposed LTXOR Features

The proposed LTXOR is the improved version of the existing LXTOR features by mapping the texton shapes into a Gaussian plane. The Gaussian plane can provide a reliable estimation of their own uncertainty. Hence, the proposed features can represent the lane with more information to assist the road/non-road classifier. In the LTXOR pattern [27], seven different texton shapes were deployed for producing texton images. Here, the image ($I_{im}$) is divided into overlapping 2×2 sub-blocks indicated by $B_{1}$. The positions of grey value are indicated by P,Q,R,S for analysis [27]. As per the texton shape, the sub-blocks are implied as in Equation (1):

(1) $$Tx\left(Y,Z\right)=\left\{\begin{array}{l} 1,\;B_{1}\left(P\right)=B_{1}\left(R\right)\&B_{1}\left(Q\right)\neq B_{1}\left(S\right)\\ 2,\;B_{1}\left(Q\right)=B_{1}\left(P\right)\&B_{1}\left(S\right)\neq B_{1}\left(R\right)\\ 3,\;B_{1}\left(R\right)=B_{1}\left(P\right)\&B_{1}\left(S\right)\neq B_{1}\left(Q\right)\\ 4,\;B_{1}\left(P\right)=B_{1}\left(Q\right)\&B_{1}\left(R\right)\neq B_{1}\left(S\right)\\ 5,\;B_{1}\left(P\right)=B_{1}\left(Q\right)\&B_{1}\left(S\right)\neq B_{1}\left(R\right)\\ 6,\;B_{1}\left(Q\right)=B_{1}\left(P\right)\&B_{1}\left(R\right)\neq B_{1}\left(S\right)\\ 7,\;B_{1}\left(P\right)=B_{1}\left(R\right)\&B_{1}\left(Q\right)=B_{1}\left(S\right)\\ 0,\;B_{1}\left(P\right)\neq B_{1}\left(R\right)\&B_{1}\left(Q\right)\neq B_{1}\left(S\right) \end{array}\right.$$

The center of every pixel and the nearby neighbors are collected on the texton picture, following the computation of the text on the image. The XOR function (⊗) is executed among the center text and neighbor. Conventionally, Equation (2) determines the LTXOR patterns [27]. As per the proposed concept, the LTXOR is modeled and updated as in Equation (3), in which $G\left(y,z\right)$ implies the Gaussian function for two dimensions, and σ implies the standard deviation, the initial value of which is taken as 0.1. σ is used for calculating the Gaussian values, those values only used to increase the LTXOR performance. In $Tx\;\left(Y,Z\right)$, x represents texton shapes, which are considered for the texton image generation; Y and Z  imply the distances from the origin to the horizontal and vertical axes, respectively. $Tx\left(b_{l}\right)\;$ and $Tx\left(b_{a}\right)\;$ imply the shape of the texton for the neighbor pixel ($b_{l}$) and center pixel ($b_{a}$), and ⊗ implies the XOR operation among the variables. The standard deviation is the distance between the horizontal and vertical:

(2) $${\mathrm{LTXOR}}_{G,L}=\overset{G}{\underset{l=1}{\sum }}2^{\left(l-1\right)}\times {\tilde{f}}_{3}\left(Tx\left(b_{l}\right)⊗Tx\left(b_{a}\right)\right)$$

(3) $${\mathrm{PLTXOR}}_{G,L}=\overset{G}{\underset{l=1}{\sum }}2^{\left(l-1\right)}\times \frac{{\tilde{f}}_{3}\left(Tx\left(b_{l}\right)⊗Tx\left(b_{a}\right)\right)}{G\left(y,z\right)}$$

(4) $$G\left(y,z\right)=\frac{1}{2\pi \sigma^{2}}e^{-\frac{y^{2}+z^{2}}{2\sigma^{2}}}$$

(5) $${\tilde{f}}_{3}\left(y⊗z\right)=\left\{\begin{array}{l} 1\;y\neq z\\ 0\;else \end{array}\right.$$

(6) $$f_{2}\left(y,z\right)=\left\{\begin{array}{l} 1\;y=z\\ 0\;else \end{array}\right.$$

Furthermore, the specific texton image is transferred to LTXOR maps with values ranging from 0 to $2^{p}-1$, where p represents the number of neighbors. The value of m is selected between 0 and $2^{p}-1$. After computing the pattern for each pixel $\left(j,k\right)$, the histogram construction can be derived, as shown in Equation (7):

(7) $$His_{PLTXORP}\left(\tilde{m}\right)=\overset{T_{1}}{\underset{\tilde{j}=1}{\sum }}\overset{T_{2}}{\underset{\tilde{k}=1}{\sum }}{\tilde{f}}_{2}\left(PLTXORP\left(j,k\right),m\right);m\in \left[0,\left(2^{p}-1\right)\right]$$

where the size of the input image is $T_{1}\times T_{2}$.

### 4.2. MTP Features

The MTP [28] combines the measurement ($I_{im}$) of the image pixels with the integration of the median. This strategy is more resistant to speckle variation (smooth or high-textured). The arithmetic means that the intensity of nine pixels is computed after a 3 × 3 neighbor is formed around each pixel. This is proven quantitatively in Equation (8), with MC,t,V implying the local median, user-specified threshold, and neighbor grey level, respectively.

(8) $$f^{MTP}=\left\{\begin{array}{ll} 1 & V>MC+t\\ 0 & MC-t\leq V\leq MC+t\\ -1 & V<MC-t \end{array}\right.$$

Every MTP code is further split into its respective negative and positive parts, which are regarded as two binary patterns known as $pos^{MTP}$ and $neg^{MTP}$. This is precisely exposed in Equations (9)–(12), where pix represents the pixel count:

(9) $$pos^{MTP}=\sum_{pix=0}^{7}f_{pos}(f_{MTP}(i_{pix}))*2^{\begin{array}{l} pix \end{array}}$$

(10) $$f_{pos}(V)=\left\{\begin{array}{cc} 1 & V=1\\ 0 & else \end{array}\right.$$

(11) $$neg^{MTP}=\sum_{pix=0}^{7}f_{neg}(f_{MTP}(i_{pix}))*2^{\begin{array}{l} pix \end{array}}$$

(12) $$f_{neg}(V)=\left\{\begin{array}{cc} 1 & V=-1\\ 0 & else \end{array}\right.$$

### 4.3. LGBPHS Features

The following approach [29] uses the local histogram feature to summarize the area attribute of the LGBP patterns. To begin, each LGBP map is separated into many non-overlapping sections. After that, each region’s histogram is retrieved. Lastly, to represent the provided image ($I_{im}$), all the histograms predicted from the areas of all the LGBP maps are combined into a unified histogram series. The following is a description of the aforementioned procedure: The histogram (H) for the image $f\left(p,q\right)$ with a grey level between [0, Ζ−1] is shown in Equation (13), wherein i implies the ith grey level, and $h_{i}$ implies the pixel count in the image with a grey level (i) [29]:

(13) $$h_{i}=\sum_{p,q}\chi \left\{f\left(p,q\right)=i\right\},\;i=0,1\ldots Z-1$$

(14) $$\chi \left(f\left(p,q\right)\right)=\left\{\begin{array}{l} 1,\;f\left(p,q\right)\;=i\\ 0,\;f\left(p,q\right)\;\neq i\; \end{array}\right.$$

At last, every histogram piece calculated from every 40 LGBP maps was combined into a histogram series, where ℜ refers to the final representation of the image, which is mentioned as $ℜ=\left(G_{0,0,0},\ldots G_{0,0,m-1},\ldots \;G_{0,1,0},\ldots \;G_{0,1,m-1},\ldots G_{7,4,m-1}\right)$ [29]. The derived feature sets, including the PLTXOR, MTP, and LGBPHS, are together appended and determined as the final feature set (fe), as in Equation (15):

(15) $$fe=\left[ℜ\;f^{MTP}\;His_{PLTXORP}\left(\tilde{m}\right)\;\right]$$

## 5. Two-Phase Classification Using BI-GRU + SI-HBO

### 5.1. Two-Phase Classification

- In Phase I, the features (fe) are subjected to BI-GRU for classification as road or non-road;
- In Phase II, the same features are then subjected to the optimized BI-GRU, which trains with the SI-HBO to determine whether they are lane or non-lane.

### 5.2. BI-GRU

The BI-GRU [30] includes unique gates called reset $\left(rt\right)$ and update $\left(ut\right)$ gates that diminish the gradient dispersal with fewer losses. The $\left(ut\right)$ substitutes the forget and input gate of LSTM and portrays the conservation degree of former data, as in Equation (16):

(16) $$ut=\mu \left(W_{u}\cdot \left[R_{t-1},\;fe_{t}\right]+f_{u}\right)$$

In Equation (16), μ points out the sigmoid activation function between 0 and 1; $fe_{t}$ stands for the input matrix at the time step (t); $R_{t-1}$ stands for the hidden state at the prior time step (t−1); $W_{u}$ stands for the weight matrix of ut; and $f_{u}$ stands for the bias matrix of ut. The $\left(rt\right)$ regulates how much chronological data have to be ignored, which is revealed in Equation (17), wherein $W_{r}$ characterizes the weight matrix of rt, and $f_{r}$ symbolizes the bias matrix of rt:

(17) $$rt=\mu \left(W_{r}\cdot \left[R_{t-1},\;fe_{t}\right]+f_{r}\right)$$

The hidden state of the candidate is exposed in Equation (18), wherein tanh stands for the tanh activation function, $f_{R}$ and $W_{R}$ stand for the bias matrix and weight matrix of the new cell state, respectively, and ∗ stands for the dot multiplication function. The output ($R_{t}$) shows linear intermission amid $\left({\tilde{R}}_{t}\right)$ and $R_{t-1}$:

(18) $${\tilde{R}}_{t}=\tanh\left(W_{R}\cdot \left[R_{t-1}\;*rg,\;fe_{t}\right]+f_{R}\right)$$

(19) $$R_{t}=\left(1-ut\right)*R_{t-1}\;+ut*{\tilde{R}}_{t}$$

The backward and forward BI-GRUs hold the prior and forthcoming details of the input data, respectively. The BI-GRU is modeled as in Equation (20). Here, ${\overset{\leftarrow }{R}}_{t}$ and ${\vec{R}}_{t}$ correspond to the hidden states of the backward and forward BI-GRUs, respectively; Ct refers to the combination of the outputs in two directions (for example, the multiplication function, average function, and summation function), where Yt is referred to as the output data:

(20) $$Yt=Ct\left({\vec{R}}_{t},\;{\overset{\leftarrow }{R}}_{t}\right)$$

### 5.3. SI-HBO Model for Tuning Bi-GRU

**Figure 2:**
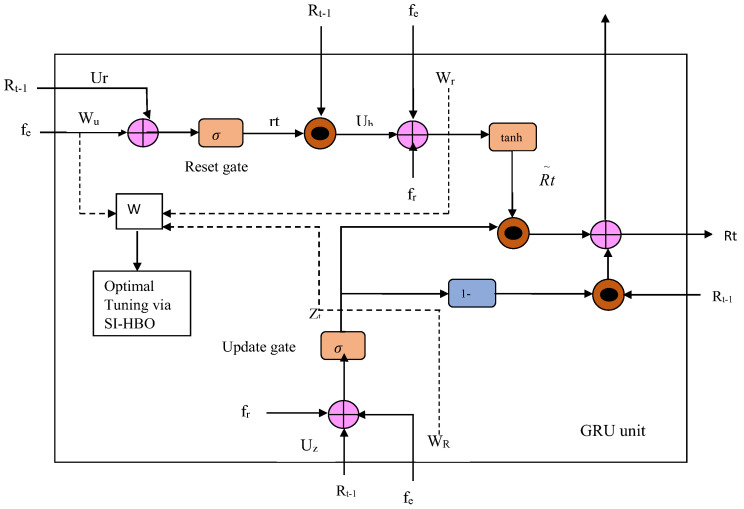
Optimal tuning of weights in Bi-GRU classifier.

The tuning of weights will be under the fixation of the objective, defined as in Equation (23), and the diagrammatic representation is shown in Figure 2. The explanation of SI-HBO is given below:

The SI-HBO approach is improved from the HBO [31] model depending on the foraging behavior of honey badgers. Self-development is better in varied optimization schemes to minimize the root mean square error, for which the improved team optimizer is used [32]. In different fields, swarm intelligence has attracted researchers [33]. The group search algorithm is nature-inspired [34]. To combine the high-level and low-level features, the semantic embedding branch is used [35]. To solve multi-objective problems, the differential evolutionary algorithm is used [36]. The coyote optimization algorithm is used for its good tracking characteristics [37]. The ring toss game-based optimization algorithm is a population-based optimization algorithm [38]. The supply–demand optimization algorithm is competitive compared to other algorithms [39]. The grasshopper optimization algorithm (GOA) is swarm-based [40]. The developed SI-HBO includes two chief phases: the digging phase and the honey phase. 

Initialization: The populations (P) are initialized, where N implies the population size and D implies the dimension. This is exposed in Equations (21) and (22):

(21) $$P=\left[\begin{array}{cccc} p_{11} & p_{12} & \ldots & p_{1D}\\ p_{21} & p_{22} & \ldots & p_{2D}\\ & \ldots \ldots \ldots \ldots \ldots \ldots \ldots \ldots \ldots & & \\ p_{N1} & p_{N2} & p_{N3} & p_{ND} \end{array}\right]$$

(22) $$p_{i}=LB_{i}+r1*\left(UB_{i}-LB_{i}\right)$$

The lower and upper limits are implied by $UB_{i}$ and $LB_{i}$, respectively. Moreover, a random number (r1) lies between 0 and 1. itr implies the current iteration, and $\max^{itr}$ implies the maximal iteration. Using Equation (23), the searching agent’s fitness is determined. 

As mentioned in Figure 2 (Phase II), the lane or non-lane classification is performed by the optimized Bi-GRU. Initially, the weight values are assigned as random values, where the weights $\left(W\right)$ that consider {$W_{u}$, $W_{r}$, and $W_{R}$} are given in Equations (16)–(18). All these weight values are given as input to the Bi-GRU. The results (output) from the Bi-GRU are considered as error values, and these values are tuned optimally by the SI-HBO algorithm. 

The major aim is to reduce the error (err) between the actual and predicted value from the Bi-GRU, which is defined in Equation (23):

(23) obj=min(err)

**Figure 3:**
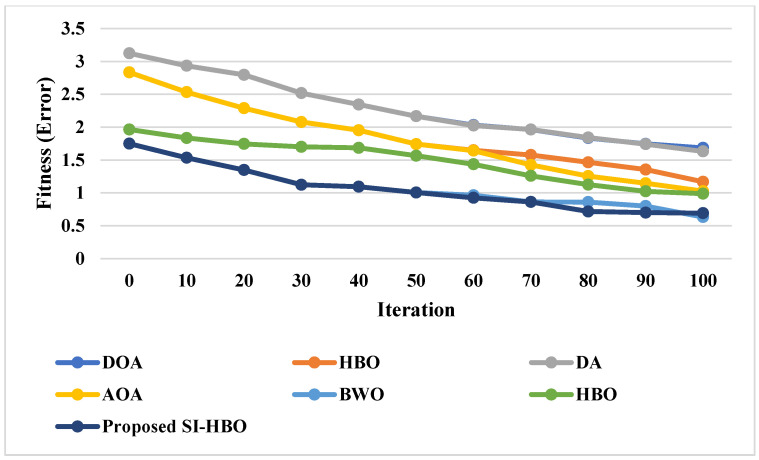
Fitness (error) vs. iteration for proposed SI-HBO.

If the error is high, then the same steps as mentioned above need to be processed; in the case of vice versa, if the error is less, then these values are tuned optimally by SI-HBO. Figure 3 shows the fitness (error values) vs. iteration for the proposed SI-HBO algorithm and other existing algorithms (DOA, DA, AOA, HBO, BWO, and HBO).

The fitness prey is referred to as $fit_{prey}$, and the best position is implied by $p_{prey}$. Ensure the termination principle: itr≤$\max^{itr}$. Use Equation (24) to update the decreasing factor (α) that minimizes by iterations to decrease randomization with time. Here, C refers to a constant, which is taken as C=2:

(24) $$\alpha =C*\exp⟮\frac{-itr}{\max^{itr}}⟯$$

For i=1  to N, compute the solution’s intensity ($I_{i}$), as in Equations (25)–(27), in which $I_{i}$ implies the prey’s scent power, and rand2 implies a random number between 0 and 1. The distance between the prey and the ith search agent is implied as $D_{i}$, whereas the source strength is implied as S:

(25) $$I_{i}=rand2*⟮\frac{S}{4\pi D_{i}^{2}}⟯$$

(26) $$S={\left(p_{itr}-p_{itr+1}\right)}^{2}$$

(27) $$D_{i}=p_{prey}-p_{i}$$

Create an arbitrary integer (r) from 0 to 1. Digging phase: If r<0.5, then update $p_{new}$ as per Equation (28), in which Sp implies the speed and time=2, and rand3, rand4, and rand5 imply random integers between 0 and 1.

(28) $$p_{new}=p_{prey}+flag*\beta *I*p_{prey}+flag*rand3*\alpha *D_{i}*Cos\left(2\pi rand4\right)*\left[1-cos(2\pi rand5\right)]$$

In the honey phase,

(29) $$p_{new}=\frac{p_{prey}+flag*\beta *I*p_{prey}+flag*rand3*\alpha *D_{i}*Cos\left(2\pi rand4\right)*\left[1-cos(2\pi rand5\right)]\;}{Sp}\;$$

(30) $$Sp=\frac{dis\tan ce}{time}$$

Here, $p_{prey}$ implies the best prey’s position and flag changes the searching direction. If 1 r≥0.5, then update the position as in Equation (31). Then, update the solution, as shown in Equation (32), in which da implies the diameter of the prey and honey badger side to side, rad implies the radius, and α implies the time-varying search influence form:

(31) $$p_{new}=p_{prey}+flag*rand7*\alpha *D_{i}$$

From Equation (31), it is detected that a honey badger executes the search near to the prey location ($p_{prey}$) depending on the distance ($D_{i}$). Now, the search behavior is inspired and varied in terms of time (α). Furthermore, a honey badger realizes some disturbance, which is eliminated by using updated Equation (32):

(32) $$p_{newupdate}=p_{prey}+\frac{flag*rand7*\alpha *D_{i}}{da}$$

(33) da=2rad

Here, rand7 lies between 0 and 1. 

Calculate a novel position and allocate it to $\;p_{newupdate}$. If $f_{new}\leq f_{i}$, then set $p_{i}$ = $p_{new}$ and $f_{i}$ = $f_{new}$, and if $f_{new}\leq f_{prey}$, then set $p_{prey}$ = $p_{new}$ and $f_{prey}$ = $f_{new}$, and execute an arithmetic crossover.

The pseudocode of the proposed algorithm is shown in Algorithm 1.
**Algorithm 1:** Pseudocode of SI-HBO algorithmInitialize the population with a random positionFitness evaluationSave best position $p_{prey}$While t ≤ tmax do                         Update the decreasing factor α using Equation (24)For i=1  to N do                         compute the solution’s intensity $I_{i}$ using Equation (25)If1 r<0.5 then                         update $p_{new}$ as per Equation (26),else                         update the solution as shown in Equation (32),End ifCalculate novel position and allocate it to $p_{newupdate}$.If $f_{new}\leq f_{i}$, & if $f_{new}\leq f_{prey}$, &$f_{prey}$ = $f_{new}$,                         execute arithmetic cross overEnd if

## 6. Results and Discussion

### 6.1. Simulation Setup

The offered road-lane-detecting scheme using (BIGRU + SI-HBO) was performed in “MATLAB 2020a” on an 11th Gen Intel(R) Core (TM) i3-1115G4 @ 3.00 GHz, 3.00 GHz, 64-bit operating system, ×64-based processor, and 8.00 GB RAM. The performance of the BI-GRU + SI-HBO method was calculated over the DOA, DA, AOA, BWO, HBO, ENet [21], LSTM, CNN [20], RNN, DBN, SVM, CNN-LD [19], RF, proposed image, and conventional image. Here, the examination was made with the database denoted as db, mentioned in [41], and named as the third lane dataset. The dataset was downloaded from https://drive.google.com/file/d/1Kisxoj7mYl1YyA_4xBKTE8GGWiNZVain/view (accessed on 6 November 2022). We can randomly generate every single modeled component, from the scene’s 3D geometry to the different object classes, according to the programmable methodology. The main road’s lane configuration is chosen. Next, we decide whether a secondary road will exist and how many lanes it will have. The secondary road junction is seen as either a merging or a split, depending on the later orientation of the camera in the image.

**Figure 4:**
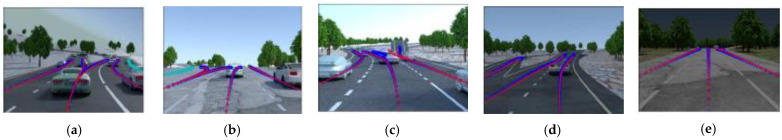
Three-dimensional sample representation of extracted lane images: (**a**) left split with divider; (**b**) straight road; (**c**) right split with divider; (**d**) road with divider; (**e**) straight road with right split.

The sample representation of the extracted lane images is shown in Figure 4.

### 6.2. Performance Analysis

**Figure 5:**
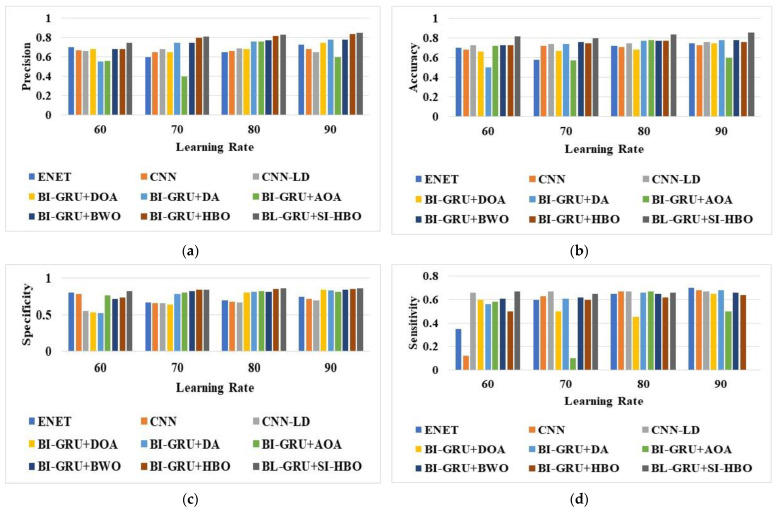
Analysis via BI-GRU + SI-HBO over other schemes ([19,20,21]) for (**a**) precision, (**b**) accuracy, (**c**) specificity, and (**d**) sensitivity for db 1.

**Figure 6:**
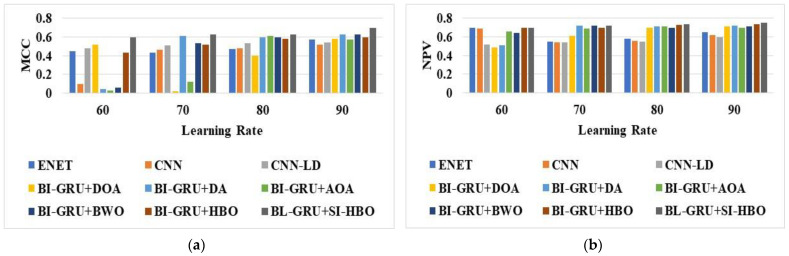

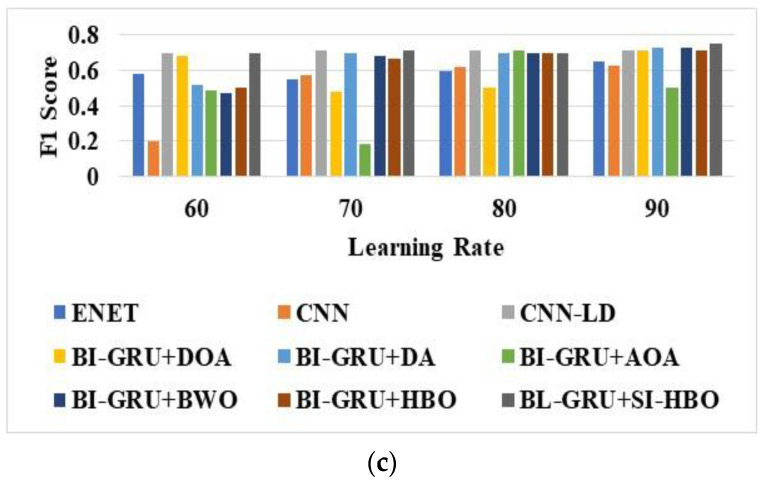
Analysis via BI-GRU + SI-HBO over other schemes ([19,20,21]) for (**a**) MCC, (**b**) NPV, and (**c**) F1-score for db 1.

**Figure 7:**
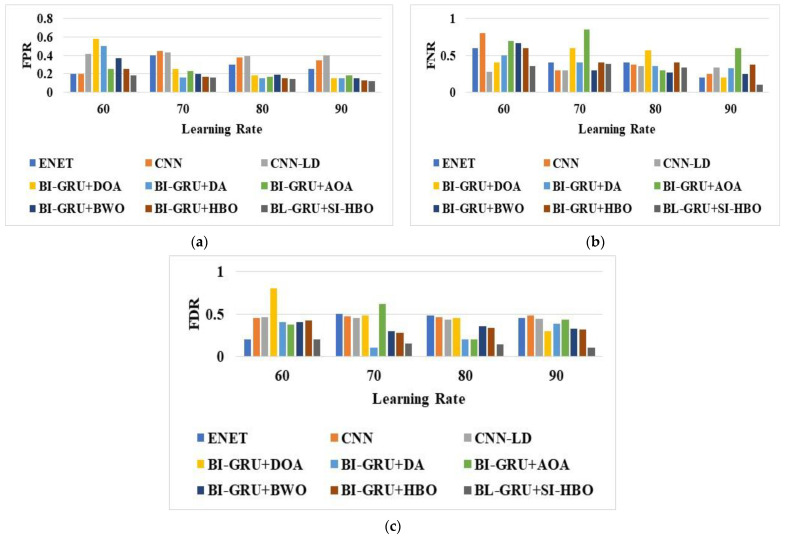
Analysis via BI-GRU + SI-HBO over other schemes ([19,20,21]) for (**a**) FPR, (**b**) FNR, and (**c**) FDR for db 1.

The analysis of the suggested BI-GRU + SI-HBO was calculated over traditional schemes on disparate metrics. The assessment of the BI-GRU + SI-HBO was performed over traditional models, such as the DOA, DA, AOA, BWO, HBO, E-Net [21], LSTM, CNN [20], CNN-LD [19], RNN, DBN, and SVM, and the RF models are presented in Figure 5, Figure 6 and Figure 7 for LRs from 60, 70, 80, and 90. Here, Figure 5, Figure 6 and Figure 7 explain the evaluation of the BI-GRU + SI-HBO over the traditional BI-GRU + DOA, BI-GRU + DA, BI-GRU + AOA, BI-GRU + BWO, and BI-GRU + HBO for db 1 to determine whether it is lane or non-lane. Table 2 explains the estimation of the BI-GRU + SI-HBO over the traditional ENet [21], LSTM, CNN [20], RNN, DBN, SVM, and RF for db 1 and db 2 to determine whether it is road or non-road. Here, the offered BI-GRU + SI-HBO offered superior outputs to the BI-GRU + DOA, BI-GRU + DA, BI-GRU + AOA, BI-GRU + BWO, BI-GRU + HBO, ENet [21], LSTM, CNN [20], RNN, DBN, SVM, and RF. In Figure 5b, the accuracy for the BI-GRU + SI-HBO is higher at the 90th LR than at the 60th, 70th, and 80th LRs. At the 60th LR, the BI-GRU + SI-HBO has a lesser accuracy than at the 70th, 80th, and 90th LRs. Likewise, from Table 2, the BI-GRU + SI-HBO had the best outcome for precision at 0.946 for db 1. Here, the accuracies for the SVM and RF are much lower, whilst the RNN has a high accuracy next to the BI-GRU + SI-HBO. This is a novel method for recognizing 3D road lanes that extracts many features, including the suggested LTXOR, LGBPHS, and MTP. Then, using an efficient BI-GRU classification procedure, it can be determined whether the object is a road or not. Then, with the aid of the SI-HBO algorithm, the optimal weights for the BI-GRU are determined. By optimally tuning the weights, precise and accurate detection is achieved. Thus, the advantage of BI-GRU + SI-HBO is established over BI-GRU + DOA, BI-GRU + DA, BI-GRU + AOA, BI-GRU + BWO, BI-GRU + HBO, E-Net [21], LSTM, CNN [20], RNN, DBN, SVM, and RF.

### 6.3. Statistical Analysis on Accuracy

Table 3 highlights the statistical study conducted via the employed BI-GRU + SI-HBO over conventional models (BI-GRU + DOA; BI-GRU + DA; BI-GRU + AOA; BI-GRU + BWO; and BI-GRU + HBO) on accuracy. The metaheuristic schemes are stochastic, and to substantiate their fair evaluation, each model was analyzed quite a lot of times to accomplish high accuracy. An accuracy of 0.928 was gained with the BI-GRU + SI-HBO for the best case, whilst the BI-GRU + DOA, BI-GRU + DA, BI-GRU + AOA, BI-GRU + BWO, and BI-GRU + HBO achieved lesser accuracies for the best case. Similarly, superior outputs were obtained for the BI-GRU + SI-HBO for the mean case. These enhancements are owing to the incorporated, enhanced LT-XOR and optimized BI-GRU concepts.

### 6.4. Comparative Analysis

**Figure 8:**
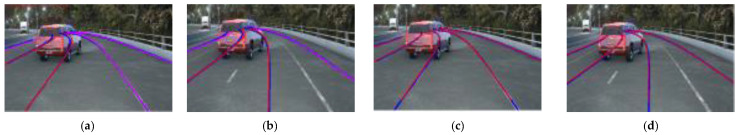
Image results of proposed and conventional road lane detection algorithms: (**a**) BI-GRU+SI-HBO; (**b**) ENet [21]; (**c**) CNN [20]; (**d**) CNN-LD [19].

An improved specificity of 0.93 is noted for the BI-GRU + SI-HBO, which is better than the BI-GRU+ SI-HBO + conventional LT-XOR, the BI-GRU+ SI-HBO without feature extraction, and the suggested method without optimization. Next to the BI-GRU + SI-HBO, the developed model without feature extraction revealed better values than the BI-GRU+ SI-HBO + conventional LT-XOR and the suggested method without optimization. This development is owing to the enhanced LT-XOR and SI-HBO concepts. The image results of the proposed and conventional methods are shown in Figure 8. From the results, it is proven that the proposed algorithm is more efficient at detecting the road and lane when compared to other methods, such as ENet [21], CNN [20], and CNN-LD [19].

The developed BI-GRU + SI-HBO technique was analyzed with the recommended scheme with conservative LT-XOR, the suggested method without feature extraction in Table 4, using db 1 and db 2. Likewise, the developed BI-GRU + SI-HBO technique is analyzed with the BI-GRU+ SI-HBO + conventional LT-XOR, the BI-GRU+ SI-HBO without feature extraction, and the suggested method without optimization in Table 5 using db 1.

### 6.5. Discussion

Currently, achieving reliability in changes in lighting and background clutter is one of the main challenges confronting researchers working on road lane detection. A number of automatic road lane detection techniques have emerged in recent years as a result of improvements in image processing techniques and the availability of low-cost visual sensing devices. The fact that lane textures can be easily distinguished from the pavement surface backdrop contributes to the viability of the automatic road lane detection method. Applying learning techniques and image processing techniques has increased the accuracy and productivity in recent years. Although these methods concentrate on identifying the lane from a single frame, they typically offer performances that are potentially unsatisfactory when dealing with some extreme circumstances, such as lane line degradation, large shadows, significant vehicle occlusion, noisy image inputs, etc. Practically, lanes should be continuous line formations on the road. As a result, information from earlier frames can be used to extrapolate the location of a lane that cannot be exactly detected in the live frame. 

From the overall analysis, research on 3D lane identification, which provides an accurate estimate of the 3D position of the drivable lanes, is becoming popular. The primary goal of this work is to propose a novel method using 3D and Phases I (road or non-road classification) and II (lane or non-lane classification). Particularly, the BI-GRU + SI-HBO obtained the greatest result for db 1 with 0.946 precision. The SVM and RF accuracies in this situation were significantly reduced; however, the RNN’s accuracy increased when compared to the BI-GRU + SI-HBO. The best-case accuracy for the BI-GRU + SI-HBO is 0.928, while the best-case accuracies for the DOA, DA, AOA, BWO, and HBO were less. Further, the analysis of the BI-GRU for the classification of road or non-road using db 1 and db 2 was performed for varied LRs. The positive metrics attained improved outcomes, while the negative metrics attained worse outcomes. From this analysis, the BI-GRU exposed a high specificity of 0.93 at the 90th LR, while at the 60th LR, the BI-GRU exposed a comparatively lesser specificity of 0.6279. Thus, better outcomes were attained at the 90th LR. Finally, the convergence of the SI-HBO scheme over the DOA, DA, AOA, BWO, and HBO for diverse iterations was analyzed in this study. From the convergence analysis, the SI-HBO gained less cost from the 10th to 50th iterations. A lesser convergence of 1.076 was accomplished using SI-HBO rather than the DOA, DA, AOA, BWO, and HBO. Thus, improved results are achieved with the SI-HBO method.

## 7. Conclusions

This work suggests a novel technique with Phase I (road or non-road classification) and Phase II (lane or non-lane classification). Initially, the features, such as the proposed LTXOR, LGBPHS, and MTP, were derived, which were then categorized via the BI-GRU, which detected whether the object was road or non-road. The similar features in a phase were then categorized via the optimal BI-GRU, wherein the weights were chosen via SI-HBO. Thus, it could be determined whether the object was lane or non-lane. The BI-GRU + SI-HBO, especially, gained the best precision at 0.946 for db 1. Here, the accuracies for the SVM and RF were much lower, whilst the RNN gained a high accuracy next to the BI-GRU + SI-HBO. An accuracy of 0.928 was gained with the BI-GRU + SI-HBO for the best case, whilst the DOA, DA, AOA, BWO, and HBO achieved lesser accuracies for the best case. In the future, effective perception via advanced methods will be proposed to study the efficient integration of sensors to minimize the computation time and cost.

## Figures and Tables

**Table 1:** Review of road-lane-detecting models.

Author	Adopted Methods	Features	Challenges
Satish et al. [19]	CNN	The method is used to extract the edge features High precision	Needs consideration of the stability and computational time analysis.
Malik et al. [20]	CNN	Less loss High recall	Needs consideration of forward collision warning policy.
Tiago et al. [21]	ENet-based model	High reliability High scalability	A special network is required for fusing purposes.
Ting et al. [22]	LCR	High detection rate Higher accuracy	Requires the use of more datasets.
Jau et al. [23]	Particle filter	Less error High accuracy	A collision-avoiding model needs to be involved.
Wang et al. [24]	Improved Hough transform	High accuracy Minimal time utilization	Cost analysis should be made.
Luo et al. [25]	Hough transform	Fewer false alarms High accuracy	Needs more consideration of time usage.
Ye et al. [26]	Gaussian distribution	High precision High F1-score	Spiral curves of the road are not considered.

**Table 2:** Analysis via BI-GRU over other classifier schemes using db 1 and db 2.

	BI-GRU + SI-HBO	ENet [21]	CNN-LD [19]	LSTM	CNN [20]	RNN	DBN	SVM	RF
Sensitivity	0.910	0.264	0.275	0.037	0.226	0.245	0.075	0.528	0.094
Accuracy	0.928	0.631	0.658	0.552	0.596	0.640	0.56	0.552	0.552
NPV	0.945	0.928	0.754	0.905	0.918	0.913	0.933	0.573	0.908
Specificity	0.945	0.930	0.983	0.725	0.918	0.903	0.903	0.573	0.910
F1-Score	0.928	0.465	0.492	0.072	0.342	0.388	0.137	0.437	0.138
FNR	0.089	0.735	0.739	0.962	0.773	0.754	0.924	0.471	0.905
Precision	0.946	0.823	0.723	0.915	0.705	0.928	0.887	0.518	0.625
FPR	0.054	0.079	0.945	0.275	0.081	0.163	0.163	0.426	0.091
MCC	0.855	0.301	0.678	0.143	0.202	0.347	0.143	0.101	0.088
FDR	0.053	0.176	0.156	0.092	0.294	0.071	0.286	0.481	0.375

**Table 3:** Statistical study on accuracy.

Metrics	Standard Deviation	Worst	Variance	Mean	Best
BI-GRU + DOA	0.108	0.580	0.011	0.655	0.816
BI-GRU + DA	0.108	0.580	0.011	0.655	0.815
BI-GRU + AOA	0.173	0.407	0.030	0.622	0.833
BI-GRU + BWO	0.088	0.647	0.007	0.767	0.855
BI-GRU + HBO	0.047	0.722	0.002	0.775	0.829
BI-GRU + SI-HBO	0.051	0.815	0.002	0.868	0.928

**Table 4:** Comparison of proposed model with conventional ones using db 1 and db 2.

Metrics	Proposed Model	Proposed Model with Conventional LTXOR	Proposed Model without Feature Extraction
Sensitivity	0.730	0.823	0.678
Accuracy	0.912	0.763	0.843
FPR	0.067	0.476	0.225
Specificity	0.932	0.523	0.733
Precision	0.919	0.679	0.755
FNR	0.269	0.172	0.321
F1-Score	0.814	0.809	0.808
FDR	0.081	0.320	0.121
MCC	0.674	0.597	0.721
NPV	0.902	0.523	0.245

**Table 5:** Comparison of the proposed model with conventional ones using db 1.

Metrics	BI-GRU + SI-HBO	BI-GRU + SI-HBO with Conventional LTXOR	BI-GRU + SI-HBO without Feature Extraction	Proposed Model without Optimization
Sensitivity	0.910	0.893	0.853	0.793
Accuracy	0.928	0.771	0.815	0.719
FPR	0.0540	0.464	0.344	0.516
Specificity	0.945	0.535	0.655	0.483
Precision	0.946	0.690	0.716	0.619
FNR	0.089	0.124	0.142	0.216
F1-Score	0.928	0.816	0.834	0.764
MCC	0.855	0.608	0.685	0.54
FDR	0.053	0.309	0.283	0.381
NPV	0.945	0.535	0.655	0.484

## Data Availability

Not applicable.

## Nomenclature

**Abbreviation**	**Description**
AOA	Arithmetic Optimization Algorithm
BWO	Black Widow Optimization
BI-GRU	Bidirectional Gated Recurrent Unit
CNN	Convolutional Neural Network
CAE	Convolution Auto-Encoder
DL	Deep Learning
DOA	Dingo Optimization
DA	Dragonfly Algorithm
DBN	Deep Belief Network
HBO	Honey Badger Optimization
HT	Hough Transform
LCR	Lane-Changing Recognition
LSTM	Long Short-Term Memory
LGBPHS	Local Gabor Binary Pattern Histogram Sequence
LTXOR	Local Texton Xor Pattern
LR	Learning Rate
MTP	Median Ternary Pattern
MLS	Mobile Laser Scanning
MCC	Matthews Correlation Coefficient
NPV	Negative Predictive Value
ROI	Region Of Interest
RNN	Recurrent Neural Network
RF	Random Forest
SVM	Support Vector Machine
SI	Self-Improved
